# On the Ethnological Characteristics of the Human Nasal Canals, Considered as an Economic Adaptation

**Published:** 1893-06

**Authors:** William C Braislin

**Affiliations:** Brooklyn, N.Y.


### SELECTIONS.

### On the Ethnological Characteristics of the Human Nasal Canals, Considered as an Economic Adaptation.

BY WILLIAM C. BRAISLIN, M.D., BROOKLYN, N.Y.

The human voice bears a constant relation to the physical construction of the voice-producing organs.

Deep, manly tones are the constant sequelæ to a larynx of large proportions, to long and slowly vibrating vocal chords; while the childish treble and the high-pitched voice of the female are the natural productions of the smaller larynx with its shorter, and, consequently, more rapidly vibrating vocal chords.

Of equal importance in the modulation, tone and indefinable individual peculiarity of the voice, is the construction of the nose and pharynx. The anatomical construction of these organs is as varying as is that of the facial features, and, to the trained eye, the individual peculiarities are as apparent.

It is not to be wondered at, therefore, that the racial peculiarities, in regard to the anatomical structures, of the internal nares are particularly striking.

The external anatomical construction of the nasal organ characteristic of the native African, is as distinguishing an ethnological differentiation as are the color of his skin, the texture of his hair, and the development of his brain.

Corresponding distinctions characterize the *internal* structure of this portion of the upper air tract of this race. Immediately on passing the anterior openings of the nares, the human nasal canals spread out into deeper and wider channels. These cavities, separated by the *septum narium*, are corrugated on their opposite surfaces by the three pairs of turbinated bodies, whose "curled-leaf" surfaces increase enormously the extent of surface area of the nasal mucous membrane.

The bones of the cranium taking part in the formation of the nasal cavities, so differ in the African race as to make these canals wider, shorter, and less deep than those of other races.

The bony ridges coursing their long axes, known as the turbinate l bones, are also more blunt and less prominent and have less of the "curled-leaf" character.

Measurements of a single specimen of an average skull of either race will suffice to show this difference in bony framework.

| | Negro. | White. |
|---|---|---|
| | Centimetres. | Centimetres. |
| Width of anterior nasal canals | 2.7 | 2.5 |
| Height of anter or nasal canals | 2.6 | 3 5 |
| Length of nasal canals (from anterior nasal spine to posterior nasal spine) | 5.2 | 5.3 |
| Length of nasal canals (from anterior nasal spine to posterior-superior angle of vomer) | 7.0 | 7.2 |
| Length of skull | 19.0 | 19.0 |
| Breadth of skull | 14.0 | 14.0 |

The continuance of these peculiarities in the lineal descendants of the negro race as seen in America to-day, is markedly striking to the student of the comparative anatomy of this region.

One, indeed, cannot fail to note in any of the clinics devoted to the diseases of this region the increased advantage which accrues to the possessor of the wider nasal canals when pathological conditions, resulting in thickening of the lining mucous membrane, attack this portion of the anatomy.

One of the most prominent American larynxologists was enabled, by means of the more patent condition of the nasal canals in his negro patients,[1]—the American descendants of this race,—

1 American Journal of the Medical Sciences, 1883.

to make a contribution of certain facts to medical science, not otherwise easily obtainable.

Mr. Wallace[2] supposes that the origin of the African race was due to a migration of some part of the race of ancestral man from the great Euro-Asiatic plateau, and that his present characteristics are the result of physiological modifications due to climatic influence.

If we accept this likely explanation in regard to the special anatomical construction of the nasal cavities of the negro and his descendants, our logical conclusion is that they must present characteristics specially adapted for preparing the inspired air of a tropical climate for reception into lung structures. We must suppose that ages of contact of his nasal mucous membrane with the atmosphere of a tropical climate have brought about, by the laws of natural selection, a nasal construction the most nearly adapted to dealing with the problem of the irritating qualities of a tropical atmosphere, of any existing race.

It does not seem probable that the evolution of the construction of the nasal canals, characteristic of the African type, has been due to any reason of better serving the purposes of the gustatory sense. The custom of regarding the nose as the organ of smell has, very properly, become modified to that of regarding it as primarily an organ of respiration. It is the proper channel for the conduction of air to the lungs for purposes of oxidation.

As has been stated, the means of warming, of moistening, and of freeing the air from dust and other irritating qualities are here most admirably afforded. The *sense of smell* must, indeed, also be regarded as a protective provision for the avoidance of substances irritating to the more delicate lung substance.

Had its purposes demanded an assistance to the procuring of food or to the avoidance of poisonous food, a development of the gustatory sense, such as is found in the dog or other animals, would have been found in the human species. This, however, is not the case. The acute sense of smell is not one highly developed or inherent in man. Indeed, this sense is soon entirely lost in conditions of the nasal canals which interfere with its respiratory function.

2 "Darwinism," P. 460. Macmillan.

The difference we have noted in the anatomical construction of this portion of the upper air tract—the nasal canals—is naturally resultant in different pathological effects in the different races as regards the particular portions of the respiratory tract most frequently becoming the seat of disease processes under the influence of atmospheric irritation. Less protection is afforded the lung structures of the negro race on account of the anatomical structure of his nasal canals, since less opposition to the irritating factors of the atmosphere is presented, by reason of the more patent and more direct course which the inspired air encounters. That the lung structure of this race in the United States suffers from the irritation caused by degenerating atmospheric conditions seems to the writer to be evinced by data of the Tenth Report of Vital Statistics of the United States, compiled by Dr. John S. Billings.

Among many others facts of great significance in this compilation we are shown that the number of deaths per 1,000 from consumption—the disease *most* dependent, perhaps, upon irritating qualities of the atmosphere—is not only greater among the colored portion of our population, but it is in direct proportion greater the more severe the rigors of climate encountered in the respective *areas of territory* from which statistical returns are cited. Thus in the Gulf coast region the proportion of deaths from consumption per one thousand deaths is about equal among the whites and blacks; but in the Middle Atlantic coast region the difference of numbers shown is a very distinct one.

In five specified areas of territory the exact proportion is as follows:—

*The Number of Deaths from Consumption in* 1,000 *Deaths from all Causes.*

| | Whites. | Colored. |
|---|---|---|
| Middle Atlantic Coast Region | 140.9 | 175.1 |
| South Atlantic Coast Region | 88.0 | 105.5 |
| Gulf Coast Region | 115 8 | 120 6 |
| The Interior Plateau | 138.4 | 126 7 |
| The Ohio River Belt | 150.7 | 2[illegible]8.1 |

Thus, while in the first case there is a difference of 34.2, in the third—the Gulf coast region —there is only a difference of a little less than 5; while in the fifth case a difference of 87.4 in the number of deaths per 1,000 from consumption exists.

We have, therefore, it seems to the writer, sufficient grounds upon which to advance the theory that the more patent condition of the nasal canals in the colored race is largely responsible for the more frequent occurrence in this race of lung disorders, as compared with the white races, in the United States.

Diseased conditions of the nasal canals in the dark race resulting in stenosis are especially rare; while, as every physician engaged in the clinical study of these disorders will testify, a condition of stenosis is one of the most common, and among the first, symptoms of disease involving this portion of the air tract in individuals of European descent.

It is unnecessary to say that the inference must not be drawn that a condition of stenosis is a safeguard against lung disorders. Any condition of the nasal passages which results in or necessitates *mouth-breathing* directly favors diseases of the lungs.

When a condition of the nasal passages exists prohibiting the free passage of air through them, the professional services of a physician or surgeon should be sought to remedy this defect. And since, as has been said, the nasal passages should be considered an integral part of the respiratory tract, the remedy should constitute not merely the rendering of this organ patent, but should also aim to restore it to a condition in which it may regain the ability of performing its normal functions of moistening, of warming, and of purifying, by freeing from irritating factors, the inspired air.

It is impossible to state how far the remedial effects of selection or evolution are modifying the peculiarities of anatomical structure of the nasal passages of the black race in the United States.

That these peculiarities become less prominent along with other racial peculiarities of the descendants of the negro race is, however, very evident to the accurate observer. To the untrained eye the external characteristics of this organ are undergoing modification, and to clinical observers a like change is noted in the internal structure; but just how far this may be due to the admixture of white blood, and how far to selective and developmental modification, is beyond the power of the writer to estimate.

